# Effects of Inhibition of Nitric Oxide Synthase on Muscular Arteries During Exercise: Nitric Oxide Does Not Contribute to Vasodilation During Exercise or in Recovery

**DOI:** 10.1161/JAHA.119.013849

**Published:** 2020-08-12

**Authors:** Kevin O'Gallagher, Husain Shabeeh, Shahzad Munir, Ali Roomi, Benyu Jiang, Antoine Guilcher, Sally Brett, Philip Chowienczyk

**Affiliations:** ^1^ Cardiovascular Division Department of Clinical Pharmacology King’s College London School of Medicine St Thomas’ Hospital London United Kingdom

**Keywords:** exercise physiology, nitric oxide, nitric oxide synthase, Vascular Biology, Physiology

## Abstract

**Background:**

Basal release of nitric oxide (NO) from the vascular endothelium regulates the tone of muscular arteries and resistance vasculature. Effects of NO on muscular arteries could be particularly important during exercise when shear stress may stimulate increased NO synthesis.

**Methods and Results:**

We investigated acute effects of NO synthase inhibition on exercise hemodynamics using N^G^‐monomethyl‐l‐arginine (l‐NMMA), a nonselective NO synthase ‐inhibitor. Healthy volunteers (n=10, 5 female, 19–33 years) participated in a 2‐phase randomized crossover study, receiving l‐NMMA (6 mg/kg, iv over 5 minutes) or placebo before bicycle exercise (25–150 W for 12 minutes). Blood pressure, cardiac output (measured by dilution of soluble and inert tracers) and femoral artery diameter were measured before, during, and after exercise. At rest, l‐NMMA reduced heart rate (by 16.2±4.3 bpm relative to placebo, *P*<0.01), increased peripheral vascular resistance (by 7.0±1.4 mmHg per L/min, *P*<0.001), mean arterial blood pressure (by 8.9±3.5 mmHg, *P*<0.05), and blunted an increase in femoral artery diameter that occurred immediately before exercise (change in diameter: 0.14±0.04 versus 0.32±0.06 mm after l‐NMMA and placebo, *P*<0.01). During/after exercise l‐NMMA had no significant effect on peripheral resistance, cardiac output, or on femoral artery diameter.

**Conclusions:**

These results suggest that NO plays little role in modulating muscular artery function during exercise but that it may mediate changes in muscular artery tone immediately before exercise.

**Nonstandard Abbreviations and Acronyms**
AI: (peripheral) augmentation index
CO: cardiac output
L‐NMMA: NG‐monomethyl‐larginine
MAP: mean arterial pressure
NO: nitric oxide
NOS: nitric oxide synthase
eNOS: endothelial nitric oxide synthase
nNOS: neuronal nitric oxide synthase

**Clinical Perspective**

**What Is New?**

- To our knowledge these are the first human data on the effect of nitric oxide synthase inhibition on the function of muscular arteries supplying exercising skeletal muscle.
- The key finding of this study is that the regulation of muscular artery tone during physiological exercise appears to be nitric oxide synthase–independent.
- Nitric oxide synthase inhibition may blunt dilation of muscular arteries supplying exercising muscle immediately before exercise but does not inhibit dilation of these arteries during or after exercise.

**What Are the Clinical Implications?**

- This study advances our understanding of the mechanisms of regulation of muscular artery function during exercise.
- This has potential implications for diseases states in which abnormalities of muscular artery tone and pressure wave reflections are thought to play a role (eg, heart failure with preserved ejection fraction).

Nitric oxide (NO) synthesized by nitric oxide synthase (NOS) within the vascular endothelium is known to regulate the tone of resistance and muscular arteries.^1^, ^2^ A transient increase in shear stress caused by increase in blood flow is a powerful stimulus to release of NO and dilation of muscular arteries (“flow‐mediated dilatation”).^3^ Sustained increases in flow also cause dilation of muscular arteries but such dilation is largely NO‐independent.^4^ Exercise causes dilation of muscular arteries supplying the exercising limb. This has important effects on systemic hemodynamics in that it reduces pressure wave reflection, reducing pressure augmentation in systole and hence load on the left ventricle.^5^, ^6^ The contribution of NO to such exercise‐induced vasodilation during which there is both a sustained increase in flow and an increase in flow pulsatility is unknown.

The purpose of the present study was to determine effects of NOS inhibition on muscular artery function during exercise using the nonselective inhibitor N^G^‐monomethyl‐l‐arginine (l‐NMMA). The direct effects of l‐NMMA on muscular arteries supplying skeletal muscle were assessed using high‐resolution ultrasound of the femoral artery before and immediately after leg exercise. In addition, a secondary objective was to confirm results of previous studies on effects of l‐NMMA on cardiac output (CO) and total peripheral resistance during exercise using pulmonary uptake of inert and soluble gas tracers.

## Methods

The data that support the findings of this study are available from the corresponding author upon reasonable request.

Subjects (n=10, 5 female) were nonsmoking healthy volunteers aged 19 to 33 years with no history of cardiovascular disease and were on no regular medication. The study comprised a 2‐phase, single‐blind, randomized controlled crossover study with subjects receiving l‐NMMA or 0.9% saline (as placebo) before exercising on 2 occasions separated by a minimum of 3 days. The study was approved by St Thomas’ Hospital research ethics committee and all subjects gave written informed consent. Studies were performed in the morning in a quiet temperature‐controlled vascular laboratory.

Subjects were instructed to avoid caffeine and exercise other than walking on the morning of the study. Following venous cannulation and after 15 minutes of rest in a semirecumbent position, baseline measurements of blood pressure, radial and digital pulse waveforms, CO, and femoral artery diameter were determined over a 15‐minute period with subjects at rest. l‐NMMA (6 mg/kg; Clinalfa, Laufelingen, Switzerland) or placebo alone was then infused over a 5‐minute period. This dose of l‐NMMA produces a marked increase in peripheral resistance at rest, which is maximal 10 to 20 minutes after infusion, the time of maximal exercise in the present protocol.^7^

Measurements of blood pressure, pulse wave indices, CO, and femoral artery diameter were repeated immediately before exercise. Subjects then performed a period of supervised exercise on a magnetically braked bicycle ergometer (Seca Cardiotest 100; Cardiokinetis, Salford, UK). Workload was automatically increased by 25 W at 2‐minute intervals from a starting workload of 25 W up to 150 W at 12 minutes. This level of exercise can be completed by the majority of recreationally active young adults.

During exercise, blood pressure, and CO were determined at 75 and 125 W. Immediately after exercise, subjects returned to a semirecumbent position. All measurements (blood pressure, radial and digital pulse waves, femoral artery diameter, and CO) were repeated immediately after exercise, and at 15 and 30 minutes after exercise. Additional femoral artery measurements were made at 5 and 10 minutes after exercise.

### Blood Pressure and Pulse Wave Measurements

At rest and after exercise, brachial systolic and diastolic blood pressure was measured by an automated oscillometric method (Omron 705CP; Omron, Japan). Radial artery pressure waveforms were obtained by applanation tonometry of the radial artery using the SphygmoCor system (Atcor, Australia) and peripheral augmentation index (peripheral AI, an index of peripheral waveform morphology that is influenced by ventricular–vascular interaction) calculated from the late systolic shoulder of the radial waveform using inbuilt software in the SphygmoCor system. During exercise, finger blood pressure was measured using a servo‐controlled finger cuff (Finometer; Finapres medical systems, the Netherlands). The SphygmoCor system cannot be used during exercise and the Finometer was therefore used to obtain recordings of the digital artery waveform from which peripheral AI was estimated as previously described.^6^ Briefly, the late systolic shoulder of the digital waveform was determined by first identifying local minima and maxima of the first derivative of the pressure signal (to identify consecutive changes in the gradient of the waveform) and then defined by the intersection of tangents to the curve at these points. This algorithm was applied to individual waveforms with a mean value of peripheral AI calculated from ≥10 individual waveforms. Values of peripheral AI identified by the algorithm were marked on the waveform and were verified by inspection of the waveform. We have previously shown that radial and digital artery waveforms obtained from the SphygmoCor and Finometer waveforms are similar^8^ and that values of peripheral AI obtained from Finometer traces are comparable to those obtained using the SphygmoCor system.^6^

### Cardiac Output

CO was determined by pulmonary uptake of soluble and inert gas tracers (InnoCor; Innovision, Scandinavia). Subjects re‐breathed a gas mixture (1% SF_6_, 5% N_2_O, and 94% O_2_) for 20‐s periods. Expired gases were sampled continuously and analyzed by an infrared photoacoustic analyzer.^9^ Stroke volume was calculated from CO and heart rate. Systemic vascular resistance was calculated from mean arterial blood pressure (MAP) and CO and expressed in units of mmHg per L/min.

### Femoral Artery Diameter

High‐resolution ultrasound (Acoustic, Aspen with 7 MHz linear probe) was used to image the femoral artery proximal to the artery bifurcation. Femoral artery diameter was measured in diastole using wall tracking software (Medical Imaging Applications, LCC, IA).

### Statistical Analysis

Data were assessed for normality using the Shapiro–Wilk test. Changes from baseline during infusion of placebo were assessed by analysis of variance for repeated measures. When this showed an overall change, values at individual time points were compared by Student paired *t* test. Changes in variables after l‐NMMA compared with placebo were assessed by 2‐way ANOVA using baseline values before l‐NMMA/placebo as the reference. All tests were 2‐sided and *P*<0.05 was taken as significant. Data are presented as mean±SEM.

## Results

Hemodynamic measurements at rest before infusion of l‐NMMA/placebo, after infusion of l‐NMMA/placebo immediately before exercise, during exercise, and during recovery are summarized in Table 1. Individual patient data for femoral artery diameter, peripheral AI, and peripheral vascular resistance can be seen in Figure S1.

**Table 1:** Hemodynamic Measurements at Rest, During Exercise, and in Recovery After Infusion of Placebo/l‐NMMA

Variable	Drug	Resting		Exercise		Recovery	
		Baseline	Postinfusion	75 W	125 W	15 min	30 min
HR, bpm	Placebo	67.2±6.2	66.9±7.0	116.8±8.0^a^	146.5±7.0^a^	76.5±6.8	69.5±5.6
	l‐NMMA	65.6±5.7	49.1±2.8^a^, ^b^, ^c^	107.7±6.6^a^, ^b^	140.9±8.2^a^	67.7±6.1^b^	69.3±6.1
SBP, mmHg	Placebo	117.4±6.2	126.9±7.5^a^	153.2±9.4^a^	166.3±8.0^a^	117.9±5.7	128.1±7.7
	l‐NMMA	120.2±4.6	138.3±4.5^a^, ^c^	152.1±6.2^a^	168.1±5.3^a^	127.4±4.3	131.6±4.7
DBP, mmHg	Placebo	69.2±4.7	74.9±4.8^a^	95.3±8.8^a^	101.0±6.2^a^	73.1±4.9	79.1±7.4
	l‐NMMA	68.9±3.6	81.9±4.4^a^	89.0±4.3^a^	99.7±4.1^a^	74.1±3.1	79.1±2.6
MAP, mmHg	Placebo	83.4±5.0	90.6±5.2^a^	113.6±8.8^a^	122.7±6.8^a^	87.3±5.0	94.5±7.8
	l‐NMMA	85.1±3.8	101.2±4.3^a^, ^b^, ^c^	110.4±4.9^a^	122.7±4.6^a^	90.7±3.3	95.1±3.0
CO, L/min	Placebo	5.1±0.4	5.1±0.4	10.6±0.5^a^	13.0±0.5^a^	5.26±0.3	4.65±0.3
	l‐NMMA	5.3±0.4	4.3±0.4^a^, ^c^	9.9±0.5^a^	12.2±0.7^a^	5.70±0.5	5.08±0.4
Stroke vol, mL	Placebo	79.2±5.1	81.0±6.8	93.8±5.9^a^	99.3±6.3^a^	73.4±7.2	71.8±6.7
	l‐NMMA	85.6±9.4	86.8±3.9	94.0±5.0	101.8±7.6	82.5±6.2	77.0±6.6
PVR, mmHg per L/min	Placebo	16.8±1.4	18.1±1.1	10.6±0.6^a^	9.2±0.4^a^	17.2±1.4	21.0±1.8^a^
	l‐NMMA	16.4±0.7	24.7±1.7^a^, ^c^	11.3±0.6^a^	9.9±0.7^a^	17.6±1.3	19.5±1.3^a^
AI, %	Placebo	53.3±5.5	44.2±5.2^a^	44.3±7.2^a^	28.6±3.1^a^	36.0±4.4^a^	42.9±6.3
	l‐NMMA	50.2±4.5	70.2±6.5^a^, ^b^, ^c^	51.0±4.3	33.5±6.4^a^	49.5±5.3^b^	48.2±4.0

AI indicates peripheral systolic augmentation index measured from the radial artery; CO, cardiac output; DBP, diastolic blood pressure; HR, heart rate; l‐NMMA, N^G^‐monomethyl‐l‐arginine; MAP, mean arterial blood pressure; PVR, peripheral vascular resistance; and SBP, systolic blood pressure.

a *P*<0.05 compared with baseline.

b *P*<0.05 compared with placebo.

c *P*<0.05 for change from baseline compared with placebo. N=10.

### Effects of l‐NMMA at Rest Before Exercise

During infusion of placebo before exercise there was no significant change in heart rate, but there was a significant increase in blood pressure, MAP increasing from 83.4±5.0 to 90.6±5.2 mmHg (*P*<0.05). Relative to placebo, l‐NMMA produced a decrease in heart rate (−16.4±3.4 versus −0.2±1.8 bpm after l‐NMMA and placebo, respectively, *P*<0.01) and an increase in peripheral resistance (8.3±1.4 versus 1.3±1.1 mmHg per L/min after l‐NMMA and placebo, respectively, *P*<0.001). There was no significant change in stroke volume, but the reduction in heart rate produced a tendency for CO to decrease after l‐NMMA. Despite this, MAP also increased after l‐NMMA relative to placebo (16.1±3.1 versus 7.2±2.2 mmHg after l‐NMMA and placebo, respectively, *P*<0.05).

Despite the increase in MAP during infusion of placebo immediately before exercise, AI decreased from 53.3±5.5 to 44.2±5.2% (*P*<0.05), accompanied by an increase in femoral artery diameter of 0.32±0.06 mm (Table 2, *P*<0.001). l‐NMMA blunted the dilation of the femoral artery (change in diameter 0.14±0.04 versus 0.32±0.06 mm after l‐NMMA and placebo, respectively, Table 2, *P*<0.01, Figure) and reversed the decrease in peripheral AI, with an increase in peripheral AI relative to that seen during infusion of placebo. This was still significant (increase of 16.8±4.3% relative to placebo, *P*<0.01) when corrected for change in heart rate, assuming a change in peripheral AI of 4% to 5% per 10 bpm change in heart rate as previously described.^6^, ^10^

**Table 2:** Femoral Artery Diameter at Baseline and in Recovery After Placebo/l‐NMMA

	Placebo		l‐NMMA	
	Diameter (mm)	Change (mm)	Diameter (mm)	Change (mm)
Baseline	7.95±0.33		8.11±0.26	
Postinfusion	8.27±0.35^a^	0.32±0.06	8.25±0.25^a^	0.14±0.04^b^
5 min postexercise	8.21±0.37^a^	0.37±0.12	8.5±0.3^a^	0.41±0.11
10 min postexercise	8.45±0.33^a^	0.50±0.11	8.5±0.3^a^	0.41±0.13
15 min postexercise	8.40±0.34^a^	0.45±0.12	8.4±0.3^a^	0.34±0.10

l‐NMMA indicates N^G^‐monomethyl‐l‐arginine.

a *P*<0.02 compared with baseline.

b *P*<0.05 compared with placebo.

**Figure 1:**
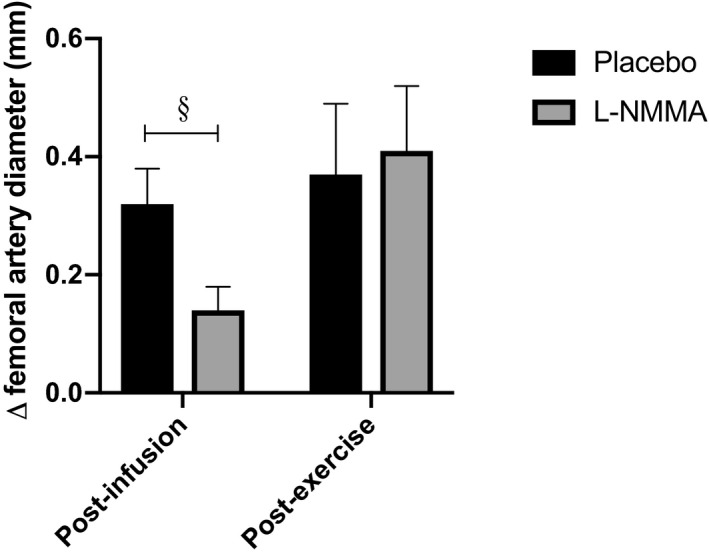
**Change in femoral artery diameter in response to infusion of either l‐NMMA or placebo.** l‐NMMA indicates N^G^‐monomethyl‐l‐arginine. Data presented as mean±SEM. ^§^ *P*<0.05 vs placebo.

### Effects of l‐NMMA During Exercise and in Recovery

All volunteers exercised for the full 12 minutes, reaching a workload of 150 W. Heart rate, CO, and MAP increased and peripheral resistance decreased during exercise, in relation to the level of workload, after both placebo and l‐NMMA. During exercise, there were no significant differences in these parameters depending upon whether l‐NMMA or placebo had been administered before exercise (Table 1). In recovery, heart rate was lower after l‐NMMA compared with placebo but CO, MAP, and peripheral resistance were similar after l‐NMMA and placebo. Peripheral AI decreased during exercise but was similar after l‐NMMA and placebo. In the recovery period, after placebo, reduction of peripheral AI relative to baseline before placebo persisted for up to 15 minutes (Table 1). After l‐NMMA, peripheral AI in recovery was higher than after placebo (49.5±5.3% versus 36.0±4.4%, *P*<0.05) but when corrected for heart rate the difference was no longer significant. Dilation of the femoral artery after placebo immediately before the start of exercise was maintained in the immediate recovery period up to 15 minutes (Table 2). By contrast to the dilation of the femoral artery occurring immediately before exercise, the sustained dilation of the femoral artery seen in the recovery period was not blunted by l‐NMMA: The mean difference between the dilatation of the femoral artery (relative to baseline) in the recovery period between placebo and l‐NMMA was −0.03 mm [−0.15, 0.08] (mean [95% CI]). The upper 95% CI is thus small in comparison to changes observed at rest.

## Discussion

This study provides insights into several aspects of the role of NOS in the physiological response to exercise in humans. The key finding is that the regulation of muscular artery tone during physiological exercise appears to be NOS‐independent.

### Effect of NOS Inhibition on Muscular Arteries

Increased flow and hence shear stress in muscular arteries is a powerful stimulus to an increase in arterial caliber. The flow‐mediated dilatation response to a transient increase in flow is mediated almost exclusively by NO.^3^ However, the response to a more sustained increase in flow is largely NO‐independent.^4^ This current study represents the first data on the effects of a sustained *physiological* increase in flow through muscular arteries supplying exercising skeletal muscle. Leg exercise was associated with dilation of the femoral artery immediately before and after exercise. However, although l‐NMMA blunted dilation of the femoral artery immediately before initiation of exercise, l‐NMMA was without effect on femoral artery diameter immediately after exercise. These data are therefore consistent with NOS‐independent mechanisms of muscular artery dilatation in response to a sustained increase in blood flow.

### Effect of NOS Inhibition on Cardiovascular Hemodynamic Properties

Previous studies examining effects of acute inhibition of NOS at rest using l‐NMMA have demonstrated a marked increase in peripheral resistance and modest increase in blood pressure.^7^, ^11^, ^12^ In the present study, measurements after infusion of l‐NMMA/placebo were made immediately before initiation of exercise and therefore may have been influenced by central command. Nevertheless, effects of l‐NMMA on peripheral resistance and blood pressure before initiation of exercise were as predicted from studies at rest: l‐NMMA produced a marked increase of 40% in peripheral resistance compared with placebo. Despite this increase in peripheral resistance, there was only a modest increase in MAP of 9 mmHg after l‐NMMA compared with placebo. Pressor effects of l‐NMMA were opposed by a reduction in CO explained by a reduction in heart rate rather than in stroke volume, which remained unchanged. Reduction in heart rate could be explained by a reflex bradycardia but is also consistent with NO regulation of sinoatrial node activity.^13^

During exercise the vasodilation of resistance vasculature supplying exercising skeletal muscle is thought to be determined by numerous “metabolic,” endothelium‐derived, and other mediators with likely redundancy of individual mediators.^14^ The contribution of NO to exercise‐induced vasodilation of resistance vasculature is relatively minor.^14^ Thus, NOS inhibition might be expected to have little influence on the decrease in peripheral vascular resistance during exercise. In the present study, despite producing a marked increase in peripheral resistance immediately before exercise, l‐NMMA had no significant effect on CO, MAP, or peripheral resistance during exercise, a finding in agreement with a previous study in which peripheral resistance was estimated from bioimpedance measurements of CO.^11^

### Effect of NOS Inhibition on Peripheral AI

AI is a complex index, influenced by ventricular–vascular interactions but may be reduced as result of a dilation of muscular arteries and reduced pressure wave reflection.^6^ l‐NMMA did not significantly block the decrease in peripheral AI during exercise and, although the decrease in peripheral AI in recovery was blunted by l‐NMMA, this was explained by a concomitant reduction in heart rate. Thus, both direct measurements of arterial diameter and measures of pressure augmentation suggest that NOS inhibition has little effect on tone of muscular arteries during exercise.

### Effect of NOS Inhibition Immediately Before Exercise

A surprising finding was that after infusion of placebo alone, there was a reduction in peripheral AI and dilation of the femoral artery immediately before exercise. These changes are likely to represent a neurally mediated response in anticipation of exercise similar to that known to exist in resistance vasculature but for which the mechanism is unknown^14^ (and which have been documented in epicardial coronary arteries^15^). l‐NMMA blunted this dilation of the femoral artery immediately before exercise and reversed the reduction in AI by an amount that could not be explained by the change in heart rate. The present study was not designed to test the complex feed‐forward regulation of tone by central command, but these findings would be consistent with blockade, by l‐NMMA, of neurally induced vasodilatation of muscular arteries immediately before exercise.

#### Limitations

Key limitations of this study include the small sample size, which restricts analysis of variation in response because of subject demographics and other characteristics and also the inability to measure femoral arterial diameter in real‐time during exercise, instead relying on postexercise measures. An additional limitation is the nonselective nature of NOS‐inhibition induced by l‐NMMA, which inhibits neuronal NOS (nNOS) in addition to endothelial NOS.^16^, ^17^ In the epicardial coronary arteries (a conduit arterial bed), it has been demonstrated that endothelial NOS and nNOS have distinct roles in the regulation of local arterial tone (ie, nNOS regulates basal arterial tone, whereas endothelial NOS is responsible for substance‐P stimulated vasodilatation).^18^ The use of a nonselective NOS‐inhibitor in this study therefore does not provide any insight into any nNOS‐dependent mechanisms in the regulation of conduit arterial tone. Further work could include assessing the differential effects of l‐NMMA compared with *S*‐methyl‐l‐thiocitrulline, an nNOS‐selective inhibitor. This would shed further light on whether the femoral arterial dilatation noted just before exercise is indeed because of nNOS‐mediated signaling, as part of neurally mediated feed‐forward mechanisms.

## Conclusions

This study suggests that in healthy subjects, inhibition of NOS is without effect on heart rate, stroke volume, or peripheral resistance during exercise. NOS inhibition appears to blunt dilation of muscular arteries supplying exercising muscle immediately before exercise but does not inhibit dilation of these arteries during or after exercise. Further studies are required to determine the influence of NO on neurally mediated changes in arterial tone in anticipation of exercise and to the NO‐independent mechanism maintaining dilation of muscular arteries during exercise.

## Sources of Funding

This work was supported by the British Heart Foundation. Dr O'Gallagher is supported by the UK Medical Research Council (Clinical Research Training Fellowship MR/R017751/1). The authors acknowledge financial support from the Department of Health via the National Institute for Health Research (NIHR) comprehensive Biomedical Research Centre award to Guy's & St Thomas’ NHS Foundation Trust in partnership with King's College London.

## Disclosures

None.

## Supporting information


**Figure S1**
