# Supplementary material for: Effects of Inhibition of Nitric Oxide Synthase on Muscular Arteries During Exercise: Nitric Oxide Does Not Contribute to Vasodilation During Exercise or in Recovery
Source: J Am Heart Assoc. 2020 Aug 12;9(16):e013849. doi: 10.1161/JAHA.119.013849 (PMC7660814; doi:10.1161/JAHA.119.013849)
Supplement: Supplementary file 1 — Figure S1 [file JAH3-9-e013849-s001.pdf]

SUPPLEMENTAL MATERIAL

Figure S1. Haemodynamic and Femoral arterial measurements at rest and in recovery after infusion of placebo/L-NMMA.

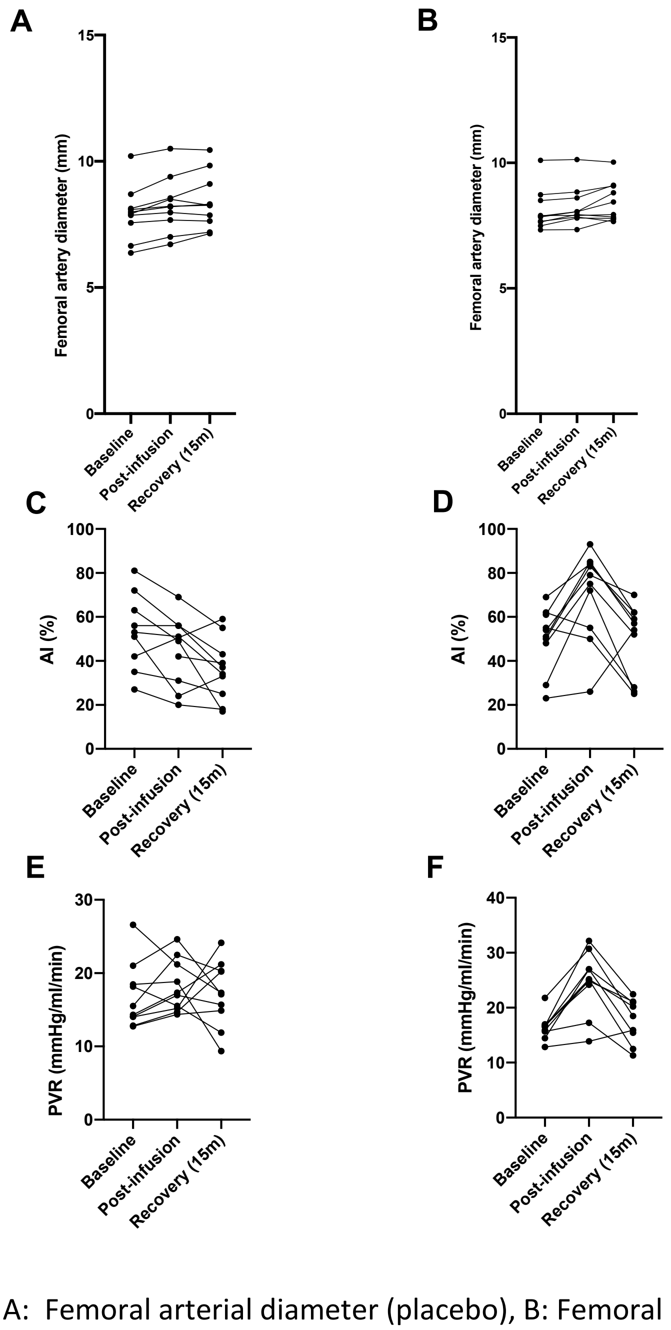

A: Femoral arterial diameter (placebo), B: Femoral arterial diameter (L-NMMA), C: Peripheral systolic augmentation index (AI, placebo), D: Peripheral systolic augmentation index (L-NMMA), E Peripheral vascular resistance (PVR, placebo), F: Peripheral vascular resistance (L-NMMA).
